# Inter-donor variability of extracellular matrix production in long-term cultures of human fibroblasts†

**DOI:** 10.1039/d1bm01933c

**Published:** 2022-06-10

**Authors:** Fabien Kawecki, Maude Gluais, Stéphane Claverol, Nathalie Dusserre, Todd McAllister, Nicolas L'Heureux

**Affiliations:** Univ. Bordeaux, Inserm, BioTis, UMR1026 F-33000 Bordeaux France nicolas.lheureux@inserm.fr; Centre de Génomique Fonctionnelle, Plateforme Protéome, University of Bordeaux F-33000 Bordeaux France; Access Medical Research Loxahatchee Florida FL USA

## Abstract

Several tissue engineering approaches are based on the ability of mesenchymal cells to endogenously synthesize an extracellular matrix (ECM) *in vitro*, which can be seen as a form of biomaterial. Accordingly, the inter-donor variability of cell-assembled extracellular matrix (CAM) production is a key parameter to understand in order to progress towards clinical applications, especially for autologous strategies. In this study, CAMs were produced, under good manufacturing process conditions, from skin fibroblasts of 21 patients as part of a clinical trial to evaluate a tissue-engineered vascular graft. The inter-donor variability of CAM strength, thickness, hydroxyproline, and glycosaminoglycan was substantial (coefficient of variability of 33%, 19%, 24%, and 19%, respectively), but a significant correlation was observed between all four properties (Pearson *r*: 0.43 to 0.70; *p*-value ≤ 0.05). A CAM matrisome analysis, performed by mass spectrometry, revealed the presence of 70 ECM-related proteins. Our study shows that the relative abundance of 16 proteins (15 non-collagenous) correlated with CAM thickness. These proteins also correlated with CAM hydroxyproline content, as well as 21 other proteins that included fibrillar collagens and non-collagenous proteins. However, data demonstrated that only the relative abundance of type I collagen subunit alpha-1 was correlated to CAM strength. This study is the most extensive evaluation of CAM inter-donor variability to date and will help tissue engineers working with this type of biomaterial to design strategies that take into account this variability, especially for autologous tissue manufacturing.

## Abstract 2

Several tissue engineering approaches are based on the ability of mesenchymal cells to endogenously synthesize an extracellular matrix (ECM) *in vitro*, which can be seen as a form of biomaterial.

## Graphical abstract

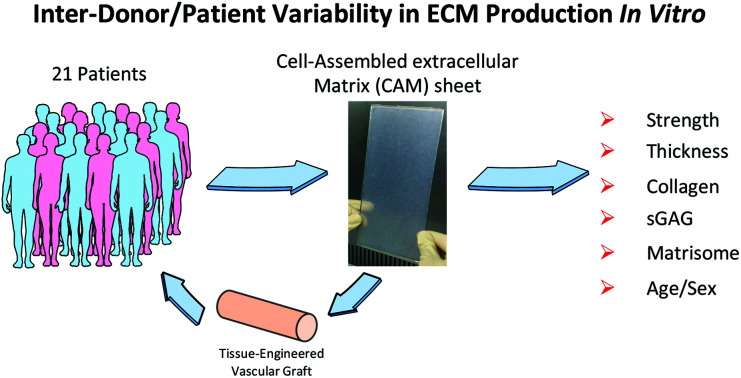

## Introduction

In the field of tissue engineering, the ideal scaffold should feature structural, physicochemical, and mechanical properties that will allow the host's body to remodel the implant in a functional tissue.^1–5^ In addition, it is important to consider the biochemical features of the matrix since they play an important role in cell-to-matrix interactions.^6^ Various biomaterials have been developed for this purpose.^7^ They can be biodegradable synthetic (*e.g.*, polylactic acid, polyglycolic acid, and polycaprolactone)^8^ or biologic (*e.g.*, chitosan, alginate, and collagen).^9^ Synthetic scaffolds can be easily produced at a low cost with great control over their composition, geometry, and structure. Nevertheless, these biodegradable materials are associated with limitations such as uncontrolled degradation rates, chronic inflammation, and a lack of biological activity.^10,11^ On the other hand, natural materials, such as collagen or fibrin gel, have the advantage of providing physiological cell-to-matrix interactions that can guide regeneration.^12,13^

Our research group focuses on a tissue engineering approach that uses Cell-Assembled extracellular Matrix (CAM) sheets for vascular applications.^14–20^ This approach relies on the ability of mesenchymal cells, such as fibroblasts, smooth muscle cells, adipose-derived stem cells, bone marrow-derived stem cells, and others, to assemble a completely biological and endogenously secreted extracellular matrix (ECM) *in vitro*.^21,22^ This CAM can be seen as a new type of biomaterial and is produced as a sheet that can be processed into different shapes such as tubes,^14–20,23^ valve leaflets,^24,25^ or yarns.^26,27^ We have pioneered the use of the human CAM for autologous and allogeneic vascular applications.^14,17,18,20^ Rolled CAM sheets were fused to produce tissue-engineered blood vessels under a good manufacturing practice (GMP) for hemodialysis access. As part of the manufacturing process, the strength and the thickness of the CAM sheets were assessed as routine quality control possibly predictive of vascular graft performance. Clinical results demonstrated that the CAM, in its tubular form, can ensure proper blood flow circulation, integrate into the native tissue, support repeated puncture, and avoid infections.

While an autologous approach has immunological advantages, manufacturing a patient-specific graft requires an understanding of the variability of cell behavior between individuals. In vascular grafts, the mechanical strength of the CAM obtained from fibroblast culture is of particular interest. To our knowledge, only one study evaluated the inter-donor variability of extracellular matrix (ECM) production. However, it compared the ECM produced after only two weeks by bone marrow-derived mesenchymal stem cells from six donors and by dermal fibroblasts from only two donors.^28^ In this study, we realized an analysis that is more comprehensive and more relevant to tissue engineering by evaluating ECM production after eight weeks of culture, by evaluating ECM mechanical strength and composition, and by including 21 donors. In addition, the tissues evaluated in this study were produced under GMP conditions using normal dermal fibroblasts obtained from an end-stage renal disease patient population requiring arteriovenous shunt for hemodialysis. Hence, these results provide a rare real-life example of patient-to-patient variability in the context of cell-based translational research.

## Materials and methods

### Human skin fibroblast extraction and expansion

All experiments were performed in accordance with the Guidelines of the *Administración Nacional de Medicamentos*, *Alimentos y Tecnología Médica* (ANMAT), and approved by the ethics committee at the *Instituto Argentino de Diagnóstico y Tratamiento* and *Instituto Nacional Central Unico Coordinador de Ablación e Implante*, or in accordance with the Guidelines of the Polish National Transplantation Council and approved by the ethics committee at the Medical University of Silesia. Informed written consent was obtained from 21 donors (10 women and 11 men; Δ age: 39 to 81 years old) for human skin fibroblast (HSF) harvests. Briefly, skin biopsies (maximum 2 cm^2^) were removed from the donor's arms using an outpatient procedure under local anesthesia. Subsequently, the dermis and epidermis were separated with thermolysin solution (25 U mL^−1^; Sigma-Aldrich, Saint-Louis, MO, USA) for two hours at 37 °C on a rocker cell culture system at 25 rock min^−1^. Human skin fibroblasts were isolated from the dermal layer using a collagenase A solution (0.40 U mL^−1^; Sigma-Aldrich) for three hours at 37 °C. The primary culture (passage 0 (P0)) of the fibroblasts was established for cellular expansion with fibroblast medium (Dulbecco–Vogt modified Eagle's medium with Ham's F12 nutrient mixture at a 3 : 1 ratio with 2.6 mM glutamine (DMEM/F-12; HyClone Laboratories Inc. – GE Healthcare Life Sciences, Logan, UT, USA) supplemented with 20% Hyclone™ fetal bovine serum III (HyClone Laboratories Inc. – GE Healthcare Life Sciences) and 50 mg mL^−1^ gentamicin (HyClone Laboratories Inc. – GE Healthcare Life Sciences) in a 37 °C humidified incubator with 5% CO_2_ and then cryopreserved. Afterward, fibroblast cells were subcultured two to five times (P2 to P5) at a density of 1 × 10^3^ to 2 × 10^4^ cell per cm^2^. All processes were realized under good manufacturing process (GMP) conditions.

### Human cell-assembled extracellular matrix sheet production

Human Cell-Assembled extracellular Matrix (CAM) sheets produced by 21 HSF populations were obtained using a clinical manufacturing context. Briefly, HSFs were seeded at a density of 1 × 10^4^ cells per cm^2^ (P3 to P6) in six-well plates and T-225 flasks (Falcon®, Thermo Fisher Scientific, San Diego, CA, USA) under GMP conditions. Cells were cultured for eight to 12 weeks with DMEM/F-12 supplemented with 20% Hyclone™ fetal bovine serum III and 500 μM sodium ascorbate (Sigma-Aldrich). The medium was changed three times per week. At the end of the culture, CAM sheets were stored at −80 °C until analysis.

### Perforation test

The strength of the fresh CAM sheets was evaluated using a perforation assay in accordance with the ISO 7198:1998/2001 standard, as previously described.^29^ The CAMs cultured within six-well plates were manually detached from the plastic and positioned on a custom-made clamping device machine. A nine-mm-diameter spherical Teflon® indenter was used to perform a perforation test. The maximal force was measured by perforating the CAMs at a constant displacement rate of 20 mm min^−1^ until rupture (*N* = 21 donors; *n* = 6 tissues from one batch/donor).

### Thickness measurements

The thickness of the CAM sheets was measured using a non-invasive technique that automatically calculates the pixel standard deviation of grayscale for the picture series taken under phase-contrast microscopy. Briefly, pictures were taken through the fresh CAMs at different focal planes at 6.6 μm-intervals. Images that were out of focus gave minimal pixel standard deviations in grayscale. As the bottom surface of the CAM came into focus, clear black lines from the cell borders became evident, and the histogram of the standard deviation increased dramatically. As the focal plane advanced above the sheet, the image became blurry again, decreasing the standard deviation. The standard deviation of the color histogram was plotted against the focal plane of the pictures. The CAM thickness was obtained by measuring the resulting curve width. The system was calibrated by measuring the known thickness of a standard glass coverslip (*N* = 21 donors; *n* = 6 tissues from one batch/donor).

### Hydroxyproline quantification

The hydroxyproline content within the CAMs of the 21 donors was determined using a hydroxyproline assay previously described.^30^ Briefly, eight-mm-diameter round samples (6.2 mm^2^) of CAM were dried at room temperature overnight. The samples were rehydrated for 30 min using 100 μL of distilled water. Subsequently, the samples were hydrolyzed with 100 μL of 10 N sodium hydroxide (NaOH; Sigma-Aldrich) at 120 °C for one hour. The hydrolysis was stopped by neutralizing the lysate with 100 μL of 10 N hydrochloride acid (HCl; Honeywell – Fluka, Seelze, Germany). The lysates and the trans-4-hydroxy-l-proline standards (Santa Cruz, Heidelberg, Germany; calibration range: 0.25, 0.5, 1, 2, 4, and 8 μg) were loaded in a 96-well plate in duplicate and dried at 65 °C for two hours. The evaporated samples and standards were incubated with 100 μL of 0.05 M Chloramine-T (Sigma-Aldrich) for 20 min. Then, the samples and standards were incubated with 100 μL of 1 M Ehrlich's solution at 65 °C for 20 min. Finally, the photocolorimetric reaction was stopped by placing the plate on ice for five min, and the absorbance was measured at 550 nm using a VICTOR multilabel plate reader (PerkinElmer, Villebon-sur-Yvette, France) (*N* = 21 donors; *n* = 6 samples from one batch/donor).

### Sulfated glycosaminoglycan assay

The sulfated glycosaminoglycan (sGAG) concentration within the CAMs of the 21 donors was determined using a Blyscan™ assay (Biocolor Ltd, Carrickfergus, Co Antrim, United Kingdom). Briefly, eight-mm-diameter round samples (6.2 mm^2^) of CAMs were dried at room temperature in thermoresistant tubes overnight. Subsequently, the samples were lyzed with 330 μL of papain extraction reagent (0.2 M sodium phosphate buffer at pH = 7.2, 0.1 M sodium acetate, 0.01 M ethylenediaminetetraacetic acid disodium salt, 5 mM l-cysteine hydrochloride, and papain crystallized suspension; Sigma-Aldrich) at 65 °C overnight. 75 μL of each tissue lysate was transferred into a new 1.5 mL cap tube and completed at 100 μL with Milli-Q water. The same Milli-Q water was used for the blank and sGAG standards were made of 1, 2, 3, 4, and 5 μg of bovine tracheal chondroitin 4-sulfate provided with the kit. One mL of dye reagent containing 1,9-dimethyl-methylene blue was added into each tube (the lysates, the blank, and the standards) and gently shaken for 30 min at 280 rpm. During this period, sGAG complexed with the dye and precipitated out from the soluble unbound dye. The tubes were centrifuged at 13 000*g* for 20 min to firmly pack the insoluble sGAG/dye complex at the bottom of the tubes. The soluble unbound dye was drained out of the tube and 0.5 mL of dissociation reagent of the kit containing the sodium salt of an anionic surfactant was added to dissolve the sGAG/dye complex into a blue-colored solution. Finally, 200 μL of each sample were loaded in a 96-well plate in duplicate, and the absorbance was measured with a 656 nm plate reader (*N* = 21 donors; *n* = 4 samples from one batch/donor).

### CAM sample preparation for liquid chromatography-tandem mass spectrometry

Eight-mm-diameter round punches of the thawed CAM were cut out and washed four times with a high salt buffer (50 mM Tris-HCl; Sigma-Aldrich), 25 mM ethylenediaminetetraacetic acid (EDTA; Sigma-Aldrich), and 3 M sodium chloride (NaCl; Sigma-Aldrich) mixed with a protease inhibitor cocktail (Sigma-Aldrich) only in the first wash (pH: 7.5). CAM samples were treated for 15 min in an extraction buffer composed of 6 M urea (Sigma-Aldrich), 2 M thiourea (Sigma-Aldrich), 100 mM ammonium bicarbonate (Sigma-Aldrich), and 10 mM 1,4-dithiothreitol (DTT; Sigma-Aldrich, pH: 7.8). The extraction buffer was then discarded, and the samples were treated based on the approach of Barrett *et al.*^31^ with 50 μL of hydroxylamine buffer (1 M hydroxylamine (Sigma-Aldrich), 4.5 M guanidine HCl (Sigma-Aldrich), and 0.2 M potassium carbonate (Sigma-Aldrich) at pH 9.0) overnight at 45 °C as previously described. Proteins were subsequently reduced with DTT, alkylated with iodoacetamide (Sigma-Aldrich), and digested with trypsin (Sigma-Aldrich, pH: 7.8) overnight at 37 °C. The samples were desalted using SepPak tC18 40 mg 96-well plates (Waters Corporation, Milford, MA, USA) according to the manufacturer's instructions. Eluates were dried and resuspended in 500 μL of water acidified with 0.1% formic acid (Sigma-Aldrich, pH: 2.5) to obtain the peptide mixture.

### Liquid chromatography-tandem mass spectrometry (LC-MS/MS) analysis

The peptide mixtures were analyzed on an Ultimate 3000 nanoLC system (Dionex, Amsterdam, Netherlands) coupled to an Electrospray Orbitrap Fusion™ Lumos™ Tribrid™ Mass Spectrometer (Thermo Fisher Scientific). Peptide digests (10 μL) were loaded onto a 300 μm-inner-diameter (ID) × 5 mm C18 PepMap™ trap column (LC Packings – Thermo Fisher Scientific) at a flow rate of 10 μL min^−1^. Peptides were eluted from the trap column onto an analytical 75 μm-ID × 50 cm Acclaim® PepMap RSLC column (LC Packings – Thermo Fisher Scientific) with a 4 to 40% linear gradient of solvent B in 45 min (solvent A was 0.1% formic acid and solvent B was 0.1% formic acid in 80% acetonitrile (ACN, 34851-1L; Sigma-Aldrich)). The separation flow rate was set at 300 nL min^−1^. The mass spectrometer operated in positive ion mode at a 2 kV needle voltage. Data were acquired using Xcalibur 4.3 software (Thermo Fisher Scientific) in a data-dependent mode. MS scans (at 375 to 1.5 × 10^3^*m*/*z*) were recorded at a resolution of *R* = 1.2 × 10^5^ (at 200 *m*/*z*) and an AGC target of 4 × 10^5^ ions collected within 0.05 s. Dynamic exclusion was set to 60 s, and top speed fragmentation in higher-energy collisional dissociation (HCD) mode was performed over a 3 s cycle. MS/MS scans with a target value of 2 × 10^3^ ions were collected in the ion trap with a maximum fill time of 35 ms. Additionally, only +2 to +7 charged ions were selected for fragmentation. Other settings were as follows: no sheath nor auxiliary gas flow, heated capillary temperature, 275 °C; normalized HCD collision energy of 35% and an isolation width of 1.6 *m*/*z*. Monoisotopic precursor selection (MIPS) was set to Peptide and the intensity threshold was set to 5 × 10^3^. Data were searched with SEQUEST HT through Proteome Discoverer 2.4 (Thermo Fisher Scientific) against the *Homo sapiens* Reference Proteome Set database (Uniprot version 2020-03; 74782 entries). Spectra from peptides higher than 5 × 10^3^ Da or lower than 350 Da were rejected. The search parameters were as follows: the mass accuracy of the monoisotopic peptide precursor and peptide fragments was set to 10 ppm and 0.6 Da, respectively. Only b- and g-ions were considered for mass calculation. Oxidation of methionines (+16 Da) and protein N-terminal modifications (Acetylation +42 Da, Met-loss −131 Da, Met-loss +Acetyl −89 Da) were considered as variable modifications and carbamidomethylation of cysteines (+57 Da) as fixed modifications. Two missed trypsin cleavages were allowed. Peptide validation was performed using the Percolator algorithm,^32^ and only “high confidence” peptides were retained, corresponding to a 1% false positive rate at the peptide level. Peaks were detected and integrated using the Minora algorithm embedded in Proteome Discoverer. Protein abundances were quantified based on the sum of all unique ion peptide signals detected for a specific protein. Quantitative data were considered for proteins quantified by a minimum of two unique peptides and a statistical *p*-value lower than 0.05 (*N* = 21 donors; *n* = 3 samples from one batch/donor).

### Statistical analyses

Data are expressed as the mean ± standard deviation (SD), and statistical analyses (graphs and Tukey box plots) were performed using the GraphPad Prism software version 8 (GraphPad Software Inc., San Diego, CA, USA). Since data showed Gaussian distributions (Shapiro–Wilk tests (alpha = 0.5) and linear QQ plots), the correlations were assessed by computing two-tailed Pearson correlation coefficients. Differences with a *p* < 0.05 were considered significant. The correlations and the heat map were graphed using GraphPad Prism version 8 and Gephi open-source software version 0.9.2 (Gephi Consortium).

## Results

### Strength, thickness, hydroxyproline content, and glycosaminoglycan quantity variability between donors

**Fig. 1:**
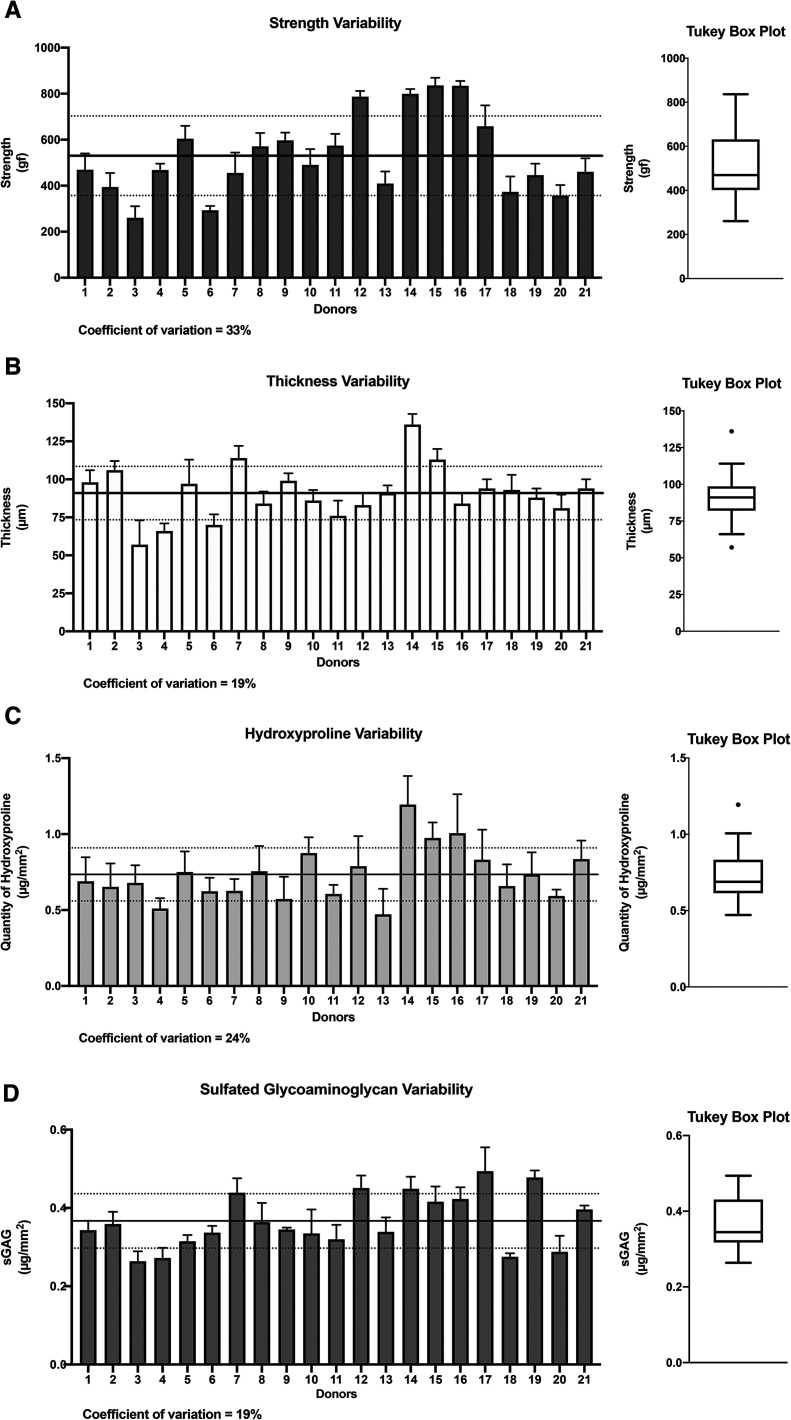
Strength, thickness, hydroxyproline, and sulfated glycosaminoglycan inter-donor variabilities. The CAMs produced by fibroblastic cell populations from 21 donors were evaluated. (A) CAM perforation strength had a mean of 530 gf (black line) and a standard deviation of ±173 gf (dotted lines) and showed the highest level of variability with a coefficient of variation of 33%. (B) CAM thickness had a mean of 91 μm (black line) and a standard deviation of ±17 μm (dotted lines) and was the more constant CAM property. (C) CAM hydroxyproline content showed a mean of 0.73 μg mm^−2^ (black line) and a standard deviation of ±0.17 μg mm^−2^ (dotted lines). (D) CAM sGAGs displayed a mean of 0.37 μg mm^−2^ (black line) and a standard deviation of ±0.07 μg mm^−2^ (dotted lines) with 19% of variability between donors. The results of each donor show the mean ± SD. Tukey box plots present the median (central black line), the interquartile range (box), the upper and lower values (“whiskers”), and the outliers (dots).

Our investigation focused on four important CAM sheet properties: perforation strength, sheet thickness, hydroxyproline content (used as a collagen content indicator^30^), and glycosaminoglycan quantity. These properties were evaluated during CAM production as a method to assess quality. The results showed moderate degrees of variability (coefficient of variability (CV%)) between donors in terms of CAM strength (33%), thickness (19%), hydroxyproline content (24%), and sulfated glycosaminoglycans (sGAGs) (19%) (Fig. 1). When considered in terms of fold, the difference between the worst and best performers was 3.2-fold for strength (min: 260 gf, max: 836 gf), 2.4-fold for thickness (min: 57 μm, max: 136 μm), 2.5-fold for hydroxyproline content (min: 0.47 μg mm^−2^, max: 1.19 μg mm^−2^), and 1.9-fold for sGAGs (min: 0.26 μg mm^−2^, max: 0.49 μg mm^−2^). Statistical evaluations, including Shapiro–Wilk tests (alpha = 0.5) and linear QQ plots, validated the normal distributions of the four data sets. However, asymmetrical distributions were observed in the case of strength, hydroxyproline content, and sGAG properties (strength: mean = 530 gf *vs.* median = 469 gf; hydroxyproline content: mean = 0.73 μg mm^−2^*vs.* median = 0.69 μg mm^−2^; and sGAGs: mean = 0.37 μg mm^−2^*vs.* median = 0.35 μg mm^−2^) but not in the case of thickness (mean = median = 91 μm). Donor 14 displayed thickness (136 μm) and hydroxyproline content (1.20 μg mm^−2^) values that were both outliers (dots on the Tukey box). Donor 3 showed a thickness value (57 μm) that was also an outlier as it was below the lower limit (so-called whisker). Taken together, these data provide a good picture of the variability of these basic values that can be expected in a real-life scenario of translational tissue engineering, despite a very controlled GMP manufacturing process.

### Correlations between the four key CAM sheet properties

**Fig. 2:**
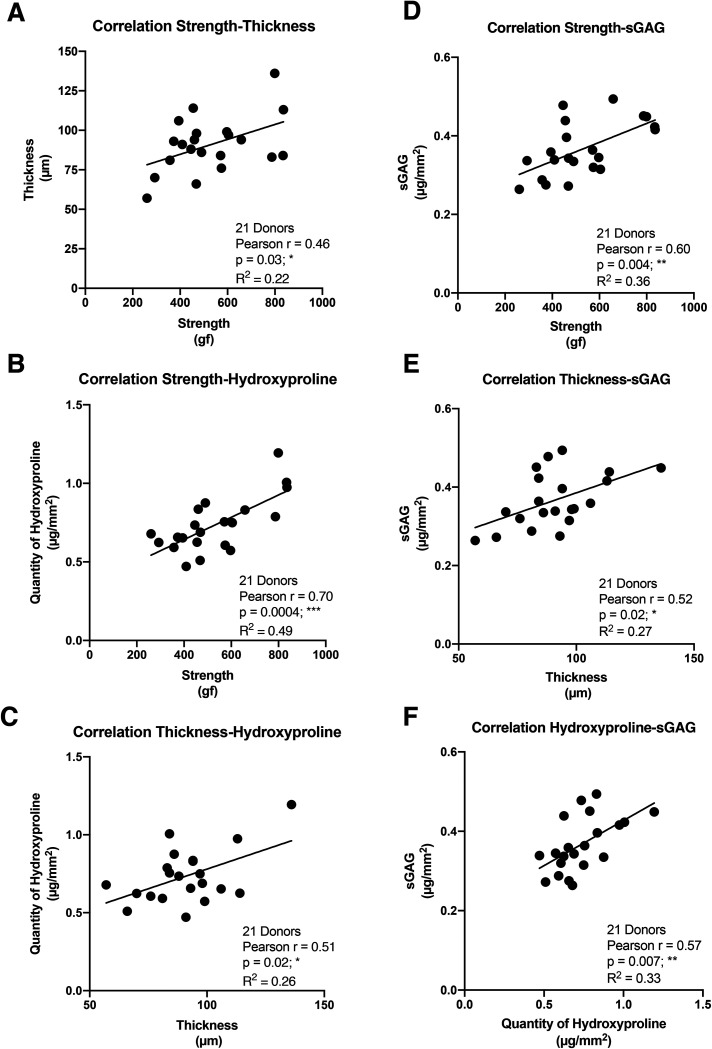
Relationships between CAM properties. (A) CAM strength displayed a moderate positive correlation with CAM thickness and (B) strongly correlated with hydroxyproline content. (C) A moderate positive correlation existed between CAM thickness and hydroxyproline content, indicating that the two properties are interrelated. (D–F) The sulfated glycosaminoglycan quantity constituting the CAM showed strong correlation with the strength property and moderate relationships with CAM thickness and hydroxyproline content.

In order to establish the strength of the relationships between the four key properties of the CAM sheets, we performed a Pearson correlation analysis, generating a correlation coefficient (Pearson *r*), which expresses how strongly two sets of paired data are linearly proportional (0 = no correlation, 1 = perfect correlation). The CAM strength displayed a statistically significant but only moderate positive correlation with the thickness (Pearson *r* = 0.46) and a strong positive correlation with the hydroxyproline content (Pearson *r* = 0.70) (Fig. 2A and B). In addition, a moderate positive connection was confirmed between the thickness and the hydroxyproline content (Pearson *r* = 0.51) (Fig. 2C). On the other hand, the CAM strength showed strong positive correlation with the quantity of sGAG constituting the CAM (Pearson *r* = 0.60) (Fig. 2D). Furthermore, sGAG quantity demonstrated moderate positive relationships with the CAM thickness (Pearson *r* = 0.52) and the hydroxyproline content (Pearson *r* = 0.57) (Fig. 2E and F). These data are consistent with the idea that the matrix organization plays an important role in CAM sheet strength and, to a lesser extent, its thickness. This also indicates that thickness is only a moderate predictor of strength.

### Correlations between the four key CAM sheet properties

#### Inventory of the CAM matrisome

The matrisome defines the ECM and ECM-related proteins composing the tissues.^28,33^ It is subdivided into the core matrisome (collagens, glycoproteins, and proteoglycans) and the matrisome-associated proteins (ECM-affiliated proteins and regulators). We performed a detailed investigation of the human CAM matrisome using liquid chromatography-tandem mass spectrometry (LC-MS/MS) by analyzing proteins that remained after an EDTA/high-salt extraction followed by a urea/thiourea extraction. A total of 70 ECM and ECM-related proteins, with at least two unique peptides, were identified within the CAM of the donors (Table 1). This included 13 collagen proteins (from seven families of collagens), 23 glycoproteins, eight proteoglycans, eight ECM-affiliated proteins, and 18 ECM regulators (Table 1). Although LC-MS/MS signal intensities cannot be compared between proteins, because each protein is detected with a different efficiency, the two highest signals were obtained for collagen type I alpha-1(I) and alpha-2(I) chains (COL1A1 and COL1A2). Periostin (POSTN) and fibrillin-1 (FBN1), as well as fibronectin (FN1) and decorin (DCN), produced the highest signals amongst glycoproteins and proteoglycans, respectively. In addition to the core matrisome, our human CAM contained ECM-affiliated proteins (*e.g.*, Annexin A2 and Hemicentin-1) and served as a reservoir for ECM regulators. While these matrisome-associated proteins generally provided a lower signal than the core matrisome proteins, some were in the same range, such as serpine H1 (SERPINH1), a chaperone protein involved in collagen biosynthesis, and matrix-remodeling-associated protein 5/adlican (MXRA5), a little known cell adhesion and matrix remodeling modulator.^34,35^

**Table 1:** Inventory of the CAM matrisome

Protein name	Gene symbol	Coverage (%)	Number of unique peptides	Protein abundance mean between donors	Standard deviation	Coefficient of variation (%)
**Collagens**						
Collagen alpha-1(I) chain	*COL1A1*	62	108	1.11 × 10^10^	8.5 × 10^9^	76
Collagen alpha-2(I) chain	*COL1A2*	63	94	9.35 × 10^9^	6.3 × 10^9^	68
Collagen alpha-1(V) chain	*COL5A1*	21	27	3.89 × 10^7^	2.4 × 10^7^	62
Collagen alpha-2(V) chain	*COL5A2*	21	28	9.15 × 10^7^	5.5 × 10^7^	60
Collagen alpha-3(V) chain	*COL5A3*	11	10	1.28 × 10^7^	9.0 × 10^6^	70
Collagen alpha-1(VI) chain	*COL6A1*	60	101	1.13 × 10^9^	9.1 × 10^8^	81
Collagen alpha-2(VI) chain	*COL6A2*	57	85	1.09 × 10^9^	8.5 × 10^8^	78
Collagen alpha-3(VI) chain	*COL6A3*	71	310	3.04 × 10^9^	2.3 × 10^9^	75
Collagen alpha-6(VI) chain	*COL6A6*	23	44	6.12 × 10^6^	7.5 × 10^6^	122
Collagen alpha-1(XII) chain	*COL12A1*	51	182	1.69 × 10^8^	1.4 × 10^8^	85
Collagen alpha-1(XIV) chain	*COL14A1*	39	57	2.90 × 10^7^	3.2 × 10^7^	110
Collagen alpha-1(XV) chain	*COL15A1*	3	4	1.37 × 10^5^	1.6 × 10^5^	116
Collagen alpha-1(XVI) chain	*COL16A1*	7	7	3.24 × 10^9^	2.4 × 10^9^	73

**Glycoproteins**						
Dermatopontin	*DPT*	63	12	9.14 × 10^6^	1.2 × 10^7^	128
EGF-containing fibulin-like extracellular matrix protein 1	*EFEMP1*	14	2	6.34 × 10^5^	4.8 × 10^5^	75
EGF-containing fibulin-like extracellular matrix protein 2	*EFEMP2*	23	3	2.38 × 10^6^	1.2 × 10^6^	51
Emilin-1	*EMILIN1*	38	54	5.57 × 10^7^	4.8 × 10^7^	86
Fibrillin-1	*FBN1*	28	84	1.66 × 10^8^	2.5 × 10^8^	150
Fibronectin	*FN1*	38	98	1.16 × 10^8^	8.7 × 10^7^	75
Fibulin-1	*FBLN1*	22	11	2.53 × 10^6^	2.5 × 10^6^	97
Fibulin-2	*FBLN2*	26	22	1.49 × 10^7^	8.9 × 10^6^	60
Fibulin-5	*FBLN5*	27	9	3.11 × 10^6^	2.0 × 10^6^	66
Laminin subunit beta-2	*LAMB2*	3	3	1.44 × 10^5^	1.3 × 10^5^	87
Latent-transforming growth factor beta-binding protein 1	*LTBP1*	5	3	4.98 × 10^5^	6.4 × 10^5^	129
Latent-transforming growth factor beta-binding protein 2	*LTBP2*	11	18	4.83 × 10^6^	5.6 × 10^6^	115
Microfibril-associated glycoprotein 4	*MFAP4*	17	2	2.38 × 10^5^	3.3 × 10^5^	140
Microfibrillar-associated protein 2	*MFAP2*	17	6	8.68 × 10^6^	1.5 × 10^7^	173
Microfibrillar-associated protein 5	*MFAP5*	34	3	2.11 × 10^6^	4.5 × 10^6^	211
Nidogen-2	*NID2*	5	3	1.29 × 10^5^	1.7 × 10^5^	131
Olfactomedin-like protein 3	*OLFML3*	17	7	6.30 × 10^5^	6.5 × 10^5^	103
Periostin	*POSTN*	68	87	1.77 × 10^8^	1.5 × 10^8^	85
Tenascin	*TNC*	49	7	4.32 × 10^7^	3.4 × 10^7^	79
Tenascin-X	*TNXB*	4	14	1.27 × 10^6^	1.2 × 10^6^	95
Thrombospondin type-1 domain-containing protein 4	*THSD4*	4	4	1.07 × 10^7^	6.9 × 10^6^	64
Thrombospondin-1	*THBS1*	32	47	4.03 × 10^7^	3.0 × 10^7^	76
Transforming growth factor-beta-induced protein ig-h3	*TGFBI*	53	58	1.20 × 10^8^	1.0 × 10^8^	85

**Proteoglycans**						
Basement membrane-specific heparan sulfate proteoglycan core protein	*HSPG2*	21	73	1.02 × 10^7^	9.6 × 10^6^	94
Biglycan	*BGN*	45	17	1.65 × 10^7^	1.3 × 10^7^	81
Decorin	*DCN*	55	31	2.96 × 10^8^	2.7 × 10^8^	90
Fibromodulin	*FMOD*	44	11	1.35 × 10^7^	1.2 × 10^7^	87
Lumican	*LUM*	53	27	5.48 × 10^7^	4.9 × 10^7^	90
Mimecan	*OGN*	29	8	1.54 × 10^6^	2.4 × 10^6^	155
Prolargin	*PRELP*	46	28	4.52 × 10^7^	4.8 × 10^7^	107
Versican	*VCAN*	10	17	5.74 × 10^6^	4.8 × 10^6^	84

**ECM-Affiliated proteins**						
Annexin A1	*ANXA1*	27	12	1.48 × 10^6^	1.3 × 10^6^	90
Annexin A2	*ANXA2*	92	48	1.81 × 10^7^	2.0 × 10^7^	111
Annexin A5	*ANXA5*	38	2	8.64 × 10^5^	7.3 × 10^5^	85
Annexin A6	*ANXA6*	16	11	9.22 × 10^5^	9.0 × 10^5^	98
Collagen triple helix repeat-containing protein 1	*CTHRC1*	9	2	2.71 × 10^5^	2.6 × 10^5^	94
Galectin-1	*LGALS1*	76	9	4.49 × 10^6^	7.3 × 10^6^	163
Galectin-3	*LGALS3*	39	10	2.82 × 10^6^	2.1 × 10^6^	75
Hemicentin-1	*HMCN1*	4	20	4.90 × 10^6^	3.3 × 10^6^	66

**ECM regulators**						
72 kDa type IV collagenase	*MMP2*	13	5	5.63 × 10^5^	5.0 × 10^5^	89
ADAMTS-like protein 4	*ADAMTSL4*	6	4	1.11 × 10^6^	8.7 × 10^5^	79
Adipocyte enhancer-binding protein 1	*AEBP1*	5	4	7.33 × 10^5^	6.7 × 10^5^	91
Cartilage intermediate layer protein 1	*CILP*	2	2	9.65 × 10^4^	1.1 × 10^5^	113
Cartilage-associated protein	*CRTAP*	7	3	2.68 × 10^5^	2.1 × 10^5^	78
Cathepsin B	*CTSB*	17	4	2.54 × 10^6^	1.9 × 10^6^	76
Glia-derived nexin	*SERPINE2*	20	4	7.10 × 10^5^	7.2 × 10^5^	102
Inter-alpha-trypsin inhibitor heavy chain H2	*ITIH2*	3	2	2.96 × 10^5^	2.6 × 10^5^	89
Lysyl oxidase homolog 1	*LOXL1*	46	16	8.12 × 10^6^	5.1 × 10^6^	63
Matrix-remodeling-associated protein 5	*MXRA5*	14	35	1.22 × 10^7^	1.2 × 10^7^	102
Pentraxin-related protein PTX3	*PTX3*	12	4	5.17 × 10^5^	8.2 × 10^5^	158
Peroxidasin homolog	*PXDN*	2	2	4.83 × 10^4^	9.3 × 10^4^	192
Prolyl 3-hydroxylase 1	*P3H1*	14	7	6.46 × 10^5^	4.4 × 10^5^	68
Prolyl 4-hydroxylase subunit alpha-1	*P4HA1*	13	4	4.06 × 10^5^	4.9 × 10^5^	120
Prolyl 4-hydroxylase subunit alpha-2	*P4HA2*	6	2	5.95 × 10^4^	6.4 × 10^4^	108
Protein-lysine 6-oxidase	*LOX*	12	4	5.07 × 10^5^	4.3 × 10^5^	84
Serine protease HTRA1	*HTRA1*	7	4	1.30 × 10^6^	9.7 × 10^5^	75
Serpin H1	*SERPINH1*	86	38	5.55 × 10^7^	4.8 × 10^7^	86

#### Inter-donor variability of the CAM matrisome

LC-MS/MS analysis revealed important inter-donor variability of protein expression. Indeed, this approach allows the comparison of the signal intensity for each protein to assess the relative protein abundance between donors (Table 1). Expression variability is quantified by the coefficient of variation for each protein. On average, proteins showed a mean coefficient of variation between donors of 97%, which is notably greater than the variations observed in CAM sheet properties. The smallest coefficient of inter-donor protein variation was 51% and was observed for glycoprotein EGF-containing fibulin-like extracellular matrix protein 2 (EFEMP2), also known as fibulin-4, which plays a key role in elastic fiber formation and binds lysyl oxidase.^36^ The highest variation was 211% for the microfibrillar-associated protein 5 (MFAP5), a component of microfibrils with other physiological roles.^37^ Variability was not correlated with signal intensity (Pearson *r* = −0.18, *p* = 0.14), confirming no detection bias.

**Fig. 3:**
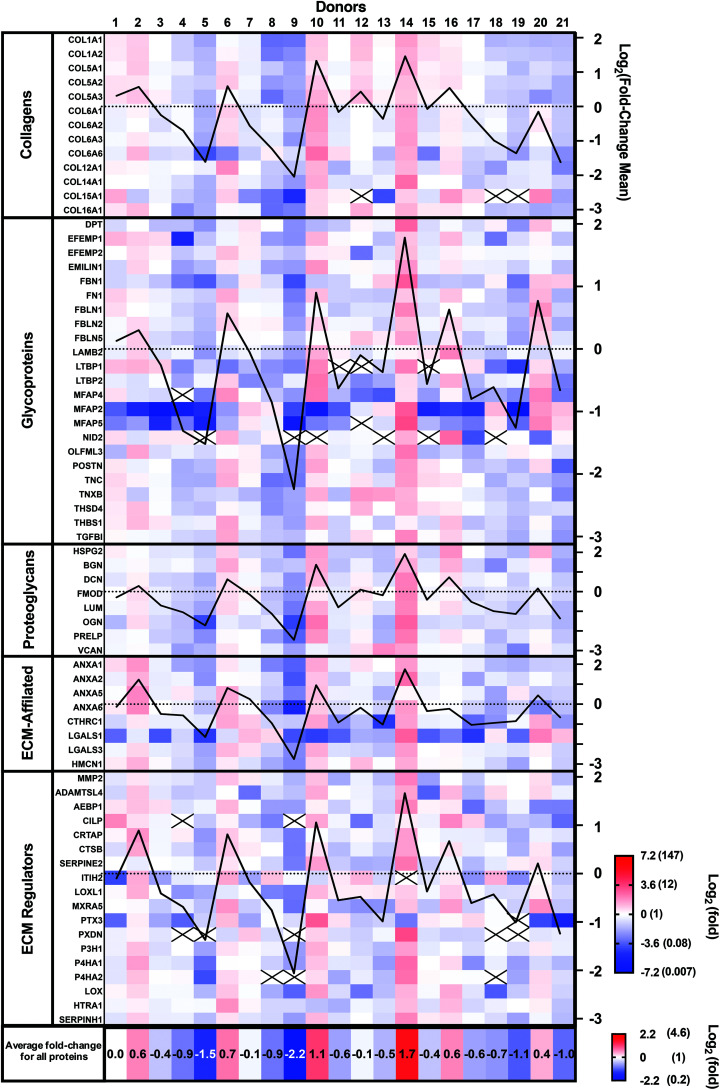
Variability of the proteomic composition of CAMs produced by human dermal fibroblasts obtained from 21 donors. Heat map of the protein abundance fold-difference from the mean of all donors using a Log_2_ color scale (−7.2 to 7.2). For each protein group, the Log_2_ of the mean fold-change was plotted (black lines, axis on the right). Mean fold-changes for all proteins are indicated for each donor at the bottom using a separate Log_2_ color scale. Data were collected from three technical replicates, and only matrisome proteins identified with at least two unique peptides were considered. Black crosses indicate proteins that were not detected.

Patient-to-patient variability and patient expression profiles can be visualized as a heat map in Fig. 3, where the colors indicate fold-differences of the abundance of each protein for each donor relative to the mean of the 21 donors using a Log_2_ scale (red: above, white: mean, and blue: below). The Log_2_ scale allows better visualization of the smaller fold-changes by avoiding the masking effect of the very large values. Protein abundance varied very widely between donors as they ranged from a Log_2_ of −7.17 (0.007-fold) to +3.21 (9.2-fold). Some donors (number 9 and 5) showed generally lower abundances, while others (number 10 and 14) demonstrated higher abundances than the mean donor level (Fig. 3). The mean fold-changes for all proteins for each patient (bottom line in Fig. 3) confirm these observations. The mean fold-change for each donor was also calculated and plotted for each protein family (black graph lines). These patient population profiles were very similar, indicating that donor abundance variations were generally observed across protein types.

Ten proteins (COL15A1, LTBP1, MFAP4 and 5, NID 2, CILP, ITIH2, PTX3, PXDN, and P4H2) were not detected in all donors. Six of these proteins accounted for 22 out of the 26 instances of lack of detection. Compared with other proteins, all 10 had relatively low signal intensity (low protein abundance), especially the group of 6, suggesting that their abundances varied around the detection threshold. In addition, 21 out of the 26 instances were observed in seven patients who had two or more proteins missing. These patients showed low mean protein fold-changes, suggesting a generally lower expression rather than downregulation of a specific protein.

The CAM composition profiles (fold-change of each protein compared to the mean abundance) of each patient are shown in the ESI (Fig. S1†). No discernable patterns appeared linked to CAM properties. So, next, we analyzed the correlation between the relative abundance of each protein and CAM properties.

### Proteins that correlate with the CAM sheet thickness

In our 3D tissue cultures, matrisome proteins accumulated by the fibroblast cells resulted in a certain matrix thickness. The relative abundance of 16 proteins correlated significantly with CAM thickness (Table 2). Surprisingly, only one collagen, the collagen alpha-1(XIV) chain (COL14A1), correlated with CAM thickness. Stronger and significant correlations were measured between the CAM thickness and several structural scaffold glycoproteins, such as FBN1, dermatopontin (DPT), and MFAP5. Some proteoglycans involved in the fibril formation rate, such as fibromodulin (FMOD) and DCN, also demonstrated a significant correlation with CAM thickness. Peroxidasin homolog (PXDN), an ECM regulator that plays a role in extracellular matrix formation,^38^ as well as prolyl 4-hydroxylase subunit alpha-1 (P4HA1), which catalyzes the post-translational formation of 4-hydroxyproline in -Xaa-Pro-Gly- sequences in collagens,^39^ showed among the highest and most significant correlations with the CAM thickness. Taken together, these data indicate that the ECM thickness correlates almost exclusively with non-collagenous proteins involved more or less directly in fibrillar collagen network assembly.

**Table 2:** List of proteins that significantly correlate with the CAM sheet thickness

Protein name	Gene symbol	Pearson *r*	*P*-Value
**Thickness *vs.* collagen**			
Collagen alpha-1(XIV) chain	*COL14A1*	0.49	0.023; *

**Thickness *vs.* glycoproteins**			
Fibrillin-1	*FBN1*	0.59	0.005; **
Dermatopontin	*DPT*	0.59	0.005; **
Microfibrillar-associated protein 5	*MFAP5*	0.55	0.010; **
Microfibrillar-associated protein 2	*MFAP2*	0.54	0.012; *
Olfactomedin-like protein 3	*OLFML3*	0.53	0.014; *

**Thickness *vs.* proteoglycans**			
Fibromodulin	*FMOD*	0.48	0.017; *
Decorin	*DCN*	0.48	0.029; *
Prolargin	*PRELP*	0.44	0.049; *
Mimecan	*OGN*	0.43	0.050; *

**Thickness *vs.* ECM-affiliated proteins**			
Annexin A2	*ANXA2*	0.55	0.009; **
Galectin-1	*LGALS1*	0.49	0.026; *

**Thickness *vs.* ECM regulators**			
Peroxidasin homolog	*PXDN*	0.63	0.002; **
Prolyl 4-hydroxylase subunit alpha-1	*P4HA1*	0.56	0.008; **
Prolyl 4-hydroxylase subunit alpha-2	*P4HA2*	0.47	0.030; *
Serpin H1	*SERPINH1*	0.47	0.036; *

### Proteins linked to the CAM sheet hydroxyproline content

Hydroxyproline is a nonessential amino acid found in the collagen proteins (approximately 10% of the amino acid mass).^40^ It is widely quantified to determine the fibrillar collagen content.^40,41^ As expected, Pearson correlation analysis showed that the CAM hydroxyproline content significantly correlated with the relative abundances of COL1A1, COL1A2, COL5A1, COL5A2, COL6A1, COL6A2, COL6A3, COL14A1, and COL16A1 (Table 3). Interestingly, CAM hydroxyproline correlated also with all 16 proteins that correlated with CAM thickness. Among these, some correlated even more strongly than type I collagens, such as DCN, prolargin, and mimecan (OGN). This group of proteoglycans closely associate together and play key roles in ECM integrity, as well as collagen fibril formation (Table 3).^42,43^ Similarly, CAM hydroxyproline showed good correlations with the abundances of SERPIN H1, as well as PXDN and prolyl 4-hydroxylase subunit alpha-1 and 2 (P4HA1 and P4HA2), which are directly involved in the collagen biosynthesis and assembly (Table 3). Fifteen non-collagenous proteins correlated with hydroxyproline but not with CAM thickness. Of these, Lumican (LUM), another proteoglycan closely associated with collagen fibrillogenesis, showed the highest correlation with CAM hydroxyproline content (Pearson *r* = 0.67; *p* < 0.001).

**Table 3:** List of proteins that significantly correlate with the CAM sheet hydroxyproline content

Protein name	Gene symbol	Pearson *r*	*P*-Value
**Hydroxyproline *vs.* collagens**			
Collagen alpha-1(XIV) chain	*COL14A1*	0.63	0.002; **
Collagen alpha-1(I) chain	*COL1A1*	0.58	0.006; **
Collagen alpha-2(I) chain	*COL1A2*	0.57	0.007; **
Collagen alpha-1(V) chain	*COL5A1*	0.54	0.012; *
Collagen alpha-3(VI) chain	*COL6A3*	0.52	0.016; *
Collagen alpha-2(V) chain	*COL5A2*	0.49	0.025; *
Collagen alpha-1(XVI) chain	*COL16A1*	0.48	0.026; *
Collagen alpha-1(VI) chain	*COL6A1*	0.48	0.029; *
Collagen alpha-2(VI) chain	*COL6A2*	0.48	0.029; *

**Hydroxyproline *vs.* glycoproteins**			
Tenascin	*TNC*	0.57	0.008; **
Emilin-1	*EMILIN1*	0.55	0.010; **
Transforming growth factor-beta-induced protein ig-h3	*TGFBI*	0.54	0.011; *
Dermatopontin	*DPT*	0.53	0.013; *
Thrombospondin type-1 domain-containing protein 4	*THSD4*	0.52	0.017; *
Fibrillin-1	*FBN1*	0.48	0.028; *
Microfibrillar-associated protein 5	*MFAP5*	0.48	0.028; *
Periostin	*POSTN*	0.47	0.030; *
Latent-transforming growth factor beta-binding protein 1	*LTBP1*	0.47	0.030; *
Laminin subunit beta-2	*LAMB2*	0.47	0.032; *
Olfactomedin-like protein 3	*OLFML3*	0.45	0.040; *

**Hydroxyproline *vs.* proteoglycans**			
Decorin	*DCN*	0.68	<0.001; ***
Prolargin	*PRELP*	0.68	<0.001; ***
Lumican	*LUM*	0.67	<0001; ***
Mimecan	*OGN*	0.61	0.004; **
Fibromodulin	*FMOD*	0.56	0.008; **
Biglycan	*BGN*	0.45	0.042; *
Hydroxyproline *vs*. ECM-Affiliated Proteins			
Annexin A2	*ANXA2*	0.56	0.009; **
Galectin-3	*LGALS3*	0.48	0.029; *

**Hydroxyproline *vs.* ECM regulators**			
Serpin H1	*SERPINH1*	0.69	<0.001; ***
Prolyl 4-hydroxylase subunit alpha-1	*P4HA1*	0.60	0.004; **
Peroxidasin homolog	*PXDN*	0.60	0.004; **
Prolyl 4-hydroxylase subunit alpha-2	*P4HA2*	0.55	0.010; **
Glia-derived nexin	*SERPINE2*	0.54	0.012; *
Adipocyte enhancer-binding protein 1	*AEBP1*	0.52	0.015; *
72 kDa type IV collagenase	*MMP2*	0.49	0.024; *
ADAMTS-like protein 4	*ADAMTSL4*	0.46	0.037; *
Protein-lysine 6-oxidase	*LOX*	0.45	0.040; *

### The role of the collagen alpha-1(I) chain within the CAM

**Fig. 4:**
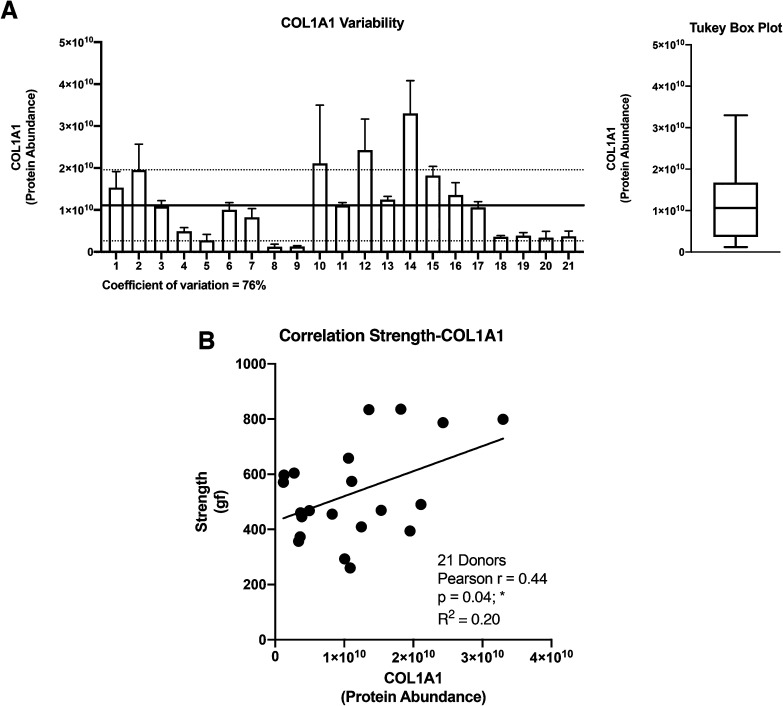
Collagen alpha-1(I) chain (COL1A1) correlation with CAM properties. (A) An elevated COL1A1 variation was measured with a global protein abundance mean of 1.1 × 10^10^ (black line) and a standard deviation of ± 8.4 × 10^9^ (dotted lines). Tukey box plot of COL1A1 abundance was realized to describe the distribution between donors. (B) COL1A1 displays a moderate positive correlation with CAM strength.

The collagen alpha-1(I) chain (COL1A1) is the most abundant subunit of type I collagen, which is the scaffold of most connective tissues.^44^ In the present study, COL1A1 provided the strongest signal in the mass spectrometry analyses, which is consistent with the remarkable strength of the CAM (Table 1). Not surprisingly, the expression profile across patients paralleled that of the alpha-2 subunit (Fig. 3). The variability of COL1A1 abundance between donors (CV = 76%, max = 3.3 × 10^10^, min = 0.12 × 10^10^, max/min = 27.5-fold) was relatively low when compared to those of other proteins but much greater than 24% observed with hydroxyproline quantification, which is typically used to quantify collagen (Table 1, Fig. 4A and 1C). The Tukey box plot showed an asymmetrical but normal distribution between donors with no outlier. Somewhat surprisingly, COL1A1 was the only ECM protein with its abundance showing correlation with CAM strength and only at a moderate level (Fig. 4B).

**Fig. 5:**
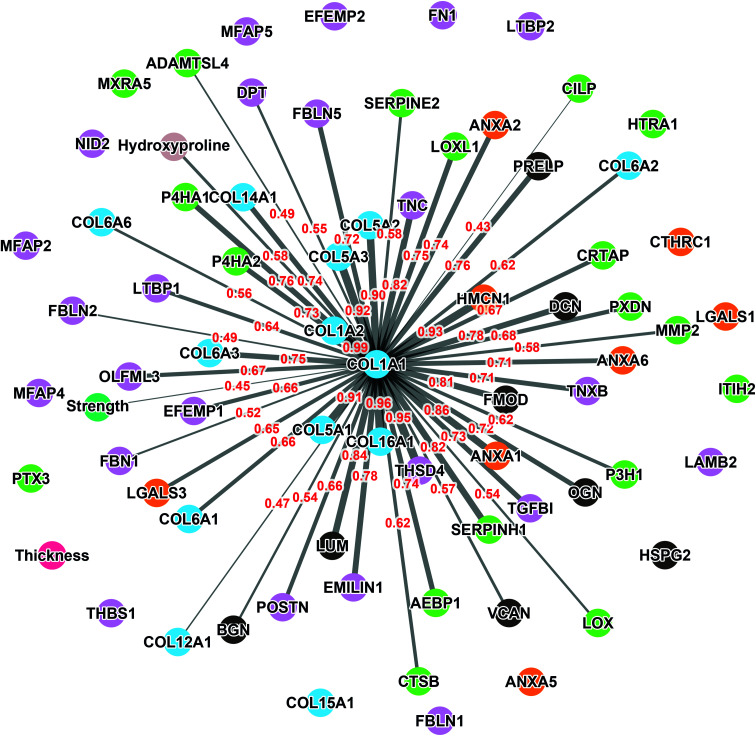
Network of correlation between COL1A1 and the detected matrisome proteins. Each colored node represents a protein (collagen proteins: bleu, glycoproteins: purple, proteoglycans: gray, ECM-affiliated proteins: orange, and ECM regulators: green) or a CAM property (strength: dark green, thickness: pink, and hydroxyproline: beige). The width of the links denotes the Pearson coefficient intensity (0.43 to 0.99; written in red) that is statistically significant (*p* < 0.05).

Correlation analysis of the relative abundance between COL1A1 and the entire matrisome indicated a significant correlation with most proteins from each family: 11/12 collagen proteins, 13/23 glycoproteins, 7/8 proteoglycans, 5/8 ECM-affiliated proteins, and 14/18 ECM regulators (Fig. 5 & the first row and first column of Fig. S2†). All the proteins that correlated with hydroxyproline were part of this group, which is consistent with the use of this assay to quantify collagen. This highlights the central role of the type I collagen network in the CAM.

As expected, COL1A1 displayed a near perfect correlation with COL1A2 and very strong relationships with type V (COL5A1, 2, and 3) and type XVI (COL16A1) collagens. Also, good correlations were observed between COL1A1 and type XIV (COL14A1) and type VI (COL6A1, 2, 3, and 6) collagens. This is consistent with the fact that all these proteins correlated significantly with the hydroxyproline content (Table 3). The highest correlations observed for non-collagenous proteins was for THSD4 (*r* = 0.95), which already correlated moderately with hydroxyproline content (*r* = 0.52), and Hemicentin-1 (HMCN1) (*r* = 0.92), a little-studied protein of the fibulin family (Fibulin-6) with roles in epithelial organization.^45–47^

### Impact of the donor age, culture passage, and sex of the donor on the ECM composition

In this study, human skin fibroblasts were extracted, for CAM production, from the arms of donors aged 39 to 81. CAMs were produced between passages three to six using the same protocol. It is well established that the donor age, anatomic donor site, or the number of passages in culture can impact the ability of cells to proliferate, differentiate, and secrete the ECM in culture.^48–50^ Our proteomic analysis confirmed that several protein abundances (*e.g.*, collagen alpha-2(VI) chain, emilin-1, prolargin, annexin A2, and ADAMTS-like protein 4) are related to donor age (Table S1†). However, data revealed that the donor age was not directly associated with any CAM properties (strength: Pearson *r* = 0.17 and *p*-value = 0.45, thickness: Pearson *r* = 0.22 and *p*-value = 0.34, and hydroxyproline content: Pearson *r* = 0.29 and *p*-value = 0.20). The culture passage showed only a negative correlation with the quantity of sGAGs within the CAM (Pearson *r* = −0.43; *p* < 0.05). In addition, a negative correlation (Pearson *r* = −0.55; **p < 0.01) was calculated for the EGF-containing fibulin-like extracellular matrix protein 2 (EFEMP2) and the culture passage. This glycoprotein, also called fibulin-4, plays a critical role in controlling collagen fibril assembly through the proteolytic activation of the lysine 6-oxidase (LOX).^51,52^ These negative correlations suggest that the GAG quantity and the abundance of EFEMP2 are reduced over passages in culture. However, the lack of correlation between age and any of the properties suggests that EFEMP2 abundance is not limiting or critical for CAM properties, which is supported by the fact that the EFEMP2 relative abundance did not correlate with any of the properties. Finally, the sex of the donors is almost equally distributed (11 men and 10 women) in this study, and no significant difference was observed in terms of strength, thickness, and hydroxyproline content (*p*-values defined using unpaired *t*-tests are 0.59, 0.71, and 0.73, respectively), suggesting that the donor's sex does not impact the quality of tissue production.

## Discussion

In the context of vascular graft tissue engineering, the mechanical properties of the extracellular matrix produced in culture have become central to many approaches.^53–55^ Our bioengineering process relies on producing a truly “bio” material we term the Cell-Assembled extracellular Matrix (CAM).^27,56–59^ The quality of this ECM, endogenously secreted and assembled by the fibroblasts of the patients themselves in culture, is critical to the success of the engineered vessel. This study sought to ascertain the inter-donor variability of the CAM produced by fibroblasts derived from 21 patients for whom tissue-engineered vascular grafts were produced as part of a clinical trial.^56,58^ This information will allow better designs of manufacturing strategies and release criteria for autologous ECM-dependent products.

In the clinical world, patient-to-patient variability is a daily fact of life. In tissue engineering research, this reality is often dismissed as reproducibility using the best finite cell population can be a challenge. In addition, obtaining cell lots from multiple donors can be complicated and, since tissue production is already complex, long, and costly, this secondary level of reproducibility is rarely addressed.

The variability of CAM strength, thickness, hydroxyproline content, and sGAG quantity seems manageable when looking at the coefficients of variation of 33%, 19%, 24%, and 19%, respectively. However, this variability appears more problematic when we look at the difference between the lower and higher values that are of 3.2-fold, 2.4-fold, 2.5-fold, and 1.9-fold, respectively.

Regarding the correlation between properties, it is surprising to see only a relatively moderate but significant correlation between CAM thickness and strength (Pearson *r* = 0.46). A variation in non-loadbearing components (sGAGs in the case here) of the matrix was identified between donors. This could indicate a different “quality” in the loadbearing elements of the matrix between donors. For example, variations in the degree of crosslinking in the structural type I collagen network would change the strength of that network (for the same volume). Conversely, it is not surprising to see a strong and very significant correlation between the hydroxyproline content and CAM strength (Pearson *r* = 0.70) since hydroxyproline is typically used as a surrogate for collagen content. Indeed, we have shown that the CAM is very rich in collagen,^59^ and the type I collagen network is well established as the main loadbearing element of conjunctive tissues (especially in the absence of elastin, as is the case here).^44^ However, the fact that this correlation is not higher could indicate, once again, that the “quality” of the type I collagen network may vary. Finally, similar to CAM strength, hydroxyproline and sGAG contents correlated only moderately with the thickness (Pearson *r* = 0.51 and 0.52, respectively). This indicates clearly that there are more factors providing the CAM than just the collagen content.

In this study, we used a proteomic approach to assess the variability of the composition of this “bio” material destined to produce autologous tissue-engineered blood vessels. Our mass spectrometry analysis revealed a complex ECM composition that are physiological components of the ECM of native organs.^33,60^ We used a more streamlined extraction protocol than in our previous study^59^ and obtained a similar protein composition but could identify more ECM proteins (70 *vs.* 56). This hydroxylamine-based digestion from Barrett *et al.* was much more reliable and experimentally less demanding than the previously used digestion.^31^ As a limitation, this protocol does not consider protein post-translational modifications since this study aims at giving a detailed protein composition rather than protein activity and function. In this study, we observed an intense protein composition variability with coefficients of variability (CV) that ranged from 51% to 221%. In some way, this variability is not surprising since Johnson *et al.* demonstrated important protein composition variability of native tissue using mass spectrometry.^61^ Indeed, the inter-donor variability between human myocardial ECMs obtained from six cadaveric donors had already seen CVs that ranged from 9% to 118%, with only six patients.^61^ It is interesting to see that the CVs of individual CAM components are much higher than those of CAM physical, mechanical, and biochemical properties. This suggests that these properties are influenced by multiple factors that temper the effects on individual proteins.

The relative expression profile was similar for each protein across the donor populations. This is more easily observed by looking at the graphed lines of Fig. 3 that show this profile for the average relative expression for each protein class. This also indicated that the CAM composition is generally similar between donors but that it is mostly the quantity (synthesis/assembly) efficacy that changes between donors.

Analysis of each patient profile for the relative abundance of individual proteins can be performed in two ways (Fig. 3 & Fig. S1†). Observation alone did not reveal the presence of a global pattern which can be associated with particular CAM properties, such as the force. A multivariable analysis may reveal some correlation between protein combination and properties and these studies are underway.

The correlation between individual proteins and CAM thickness revealed that 16 proteins showed significant correlation at moderate to almost strong levels with the Pearson *r* ranging from 0.43 to 0.63 (Table 2). Notably, type I collagen subunits were not in that group, nor were there other high-signal collagens. This is consistent with the only moderate correlation observed between the CAM thickness and strength or hydroxyproline content. These non-loadbearing proteins could participate significantly in the CAM volume, which would lower the thickness–hydroxyproline correlation. Also, this group includes proteins that influence fibrillogenesis (*e.g.*, dermatopontin, fibromodulin, and decorin) and hydroxylation (prolyl 4-hydroxylase) of collagen.^42,62,63^ Hence, these proteins could also impact the “quality” of the collagen network causing it to be less dense or less efficiently loadbearing for the same volume; both would reduce the correlation between the CAM thickness and strength or hydroxyproline content.

The correlation between individual proteins and CAM hydroxyproline content revealed a much larger group of proteins (*i.e.*, 37) with significant and fairly high correlation Pearson *r* values ranging from 0.45 to 0.69. Not surprisingly, this includes fibrillar and fibril-associated collagens. All other proteins are linked, more or less directly, to collagen synthesis, fibrillogenesis, or assembly, or simply bind to fibrillar collagen. Finally, it should be noted that almost all the proteins that correlate with CAM thickness (Table 2) also correlate with hydroxyproline content (Table 3), which is consistent with the fact that the CAM sheet thickness and hydroxyproline content were correlated (Fig. 2).

The relative abundance of type I collagen (subunit alpha-1) correlated significantly with 50 of the 70 ECM proteins identified in the CAM. Of those, 27 correlations had a Pearson *r* ≥ 0.70, indicating a strong relationship. All these proteins have established roles in fibrillar collagen network synthesis, assembly, or stability. Surprisingly, COL1A1 was the only protein to significantly correlate with the puncture strength of the CAM. While this is consistent with the unique mechanical role of type I collagen, this finding was surprising since we would have expected many of the high correlations between some proteins and COL1A1 to translate into a correlation with force. This is likely due to the fairly low correlation between COL1A1 and force (Pearson *r* = 0.44), which was even lower than the correlation between the biochemically measured collagen (hydroxyproline) and strength (*r* = 0.51). As mentioned before, these results show that the strength of the collagen network depends on one or more factors other than collagen content, which might control such variables as 3D network organization and crosslinking density or type. In addition, data did not reveal a direct correlation between CAM strength and LOX abundance detected by mass spectrometry (Pearson *r* = 0.07, *p* = 0.78, non-significant). This may appear surprising since LOX is responsible for converting lysine molecules into highly reactive aldehydes that form crosslinks in collagen molecules, which increases collagen strength. However, since LOX is an enzyme, its activity may not increase due to an accumulation phenomenon as in the case with structural proteins. In addition, the activity and concentration, over the eight weeks of culture, may have varied widely depending on various factors (*e.g.*, pH, co-factors, and cell phenotype/activity). These results also suggest that its role in crosslinking matrix proteins is more dependent on its activity than on its quantity. An interesting study would be to compare the degree of collagen crosslinking and the relative level of LOX to see if the concentration of the enzyme and the end of the CAM production cycle are correlated with collagen crosslinking. The lack of correlation of other proteins with strength was disappointing since we had hoped to identify potential initiators or bottlenecks of collagen network assembly. While correlated abundances could have been likely due to the consequence of abundant collagen (*e.g.*, associated proteins), some correlations could have given new targets to promote collagen network assembly to accelerate the production of ECM-dependent tissue-engineered constructs.

## Conclusions

After the manufacturing cost, inflated by ill-adapted regulatory requirements, the inter-donor variability is the most important challenge for clinical tissue engineering. In this study, we quantified this variability in terms of ECM assembly, the most “bio” of all biomaterials. This variability was very significant in tissue thickness, strength, collagen content, and glycosaminoglycan quantity. While correlated, the relationship between these properties was not as simple as one could expect and suggests a complex multifactorial regulation. Matrisome analysis showed an even more intense variability when individual proteins are considered. However, protein expression profiles suggest that, despite large differences in the levels of ECM assembly, the CAMs from different donors have similar compositions. While donor-to-donor variability can be managed with a well-designed manufacturing process and relevant release criteria, these data support an allogeneic approach, when appropriate, to minimize the impact of this variability.

## Author contributions

Fabien Kawecki: Conceptualization, methodology, validation, formal analysis, data curation, investigation, writing – original draft, visualization, and funding acquisition. Maude Gluais: Writing – review & editing, methodology, validation, formal analysis, investigation, and data curation. Stéphane Claverol: Methodology, validation, formal analysis, investigation, and resources. Nathalie Dusserre: Resources and methodology. Todd McAllister: Resource and methodology. Nicolas L'Heureux: Conceptualization, methodology, formal analysis, resources, writing – review & editing, supervision, project administration, and funding acquisition.

## Conflicts of interest

The authors declare that they have no known competing financial interests or personal relationships that could have influenced the work reported in this paper.

## Supplementary Material

BM-010-D1BM01933C-s001
