# Supplementary material for: Inter-donor variability of extracellular matrix production in long-term cultures of human fibroblasts
Source: Biomater Sci. 2022 Jun 10;10(14):3935–50. doi: 10.1039/d1bm01933c (PMC9275472; doi:10.1039/d1bm01933c)
Supplement: BM-010-D1BM01933C-s001 [file BM-010-D1BM01933C-s001.pdf]

Electronic Supplementary Material (ESI) for Biomaterials Science.
This journal is © The Royal Society of Chemistry 2022

Supplementary Materials

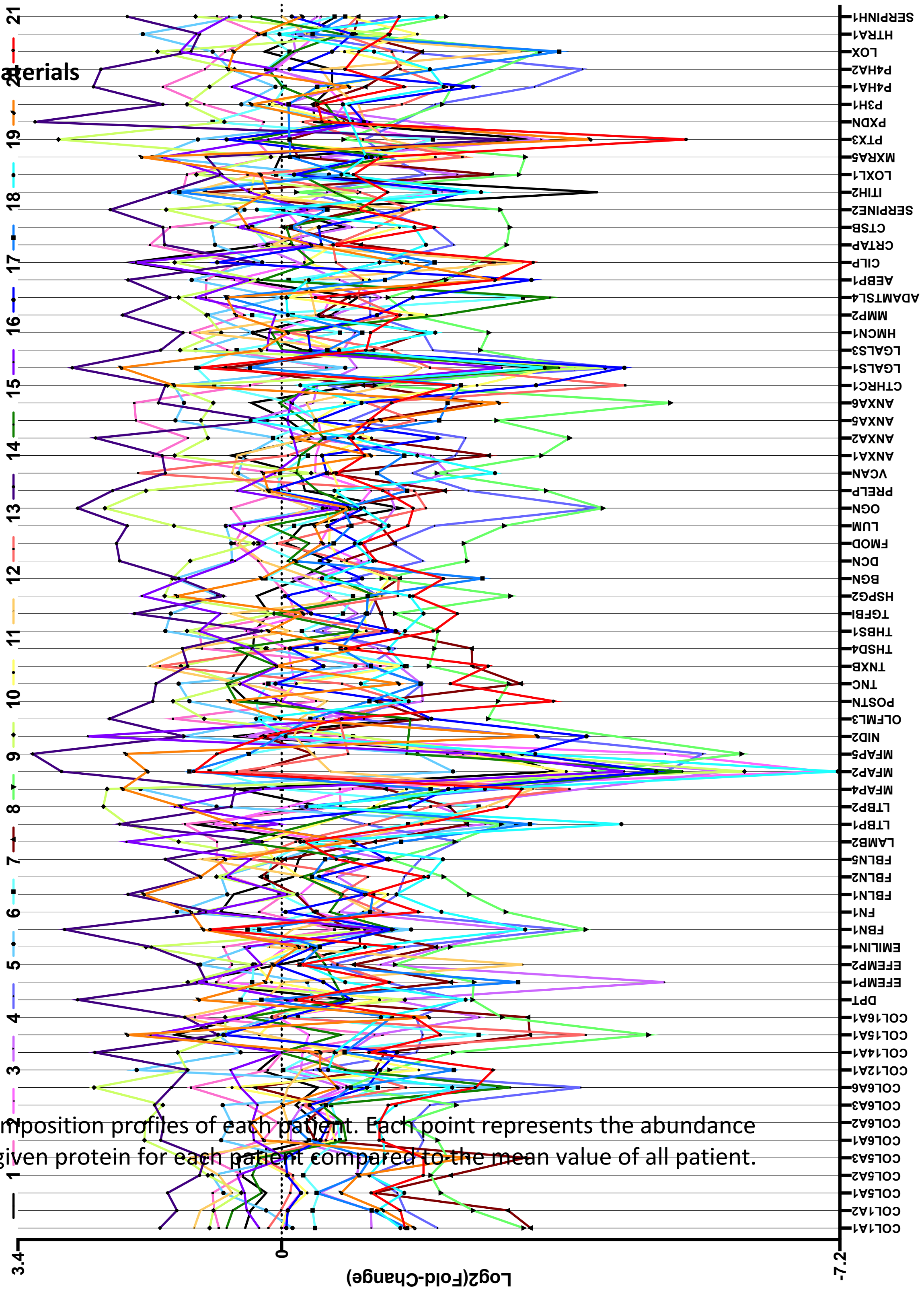

Figure S1. CAM composition profiles of each patient. Each point represents the abundance fold-change for a given protein for each patient compared to the mean value of all patient.

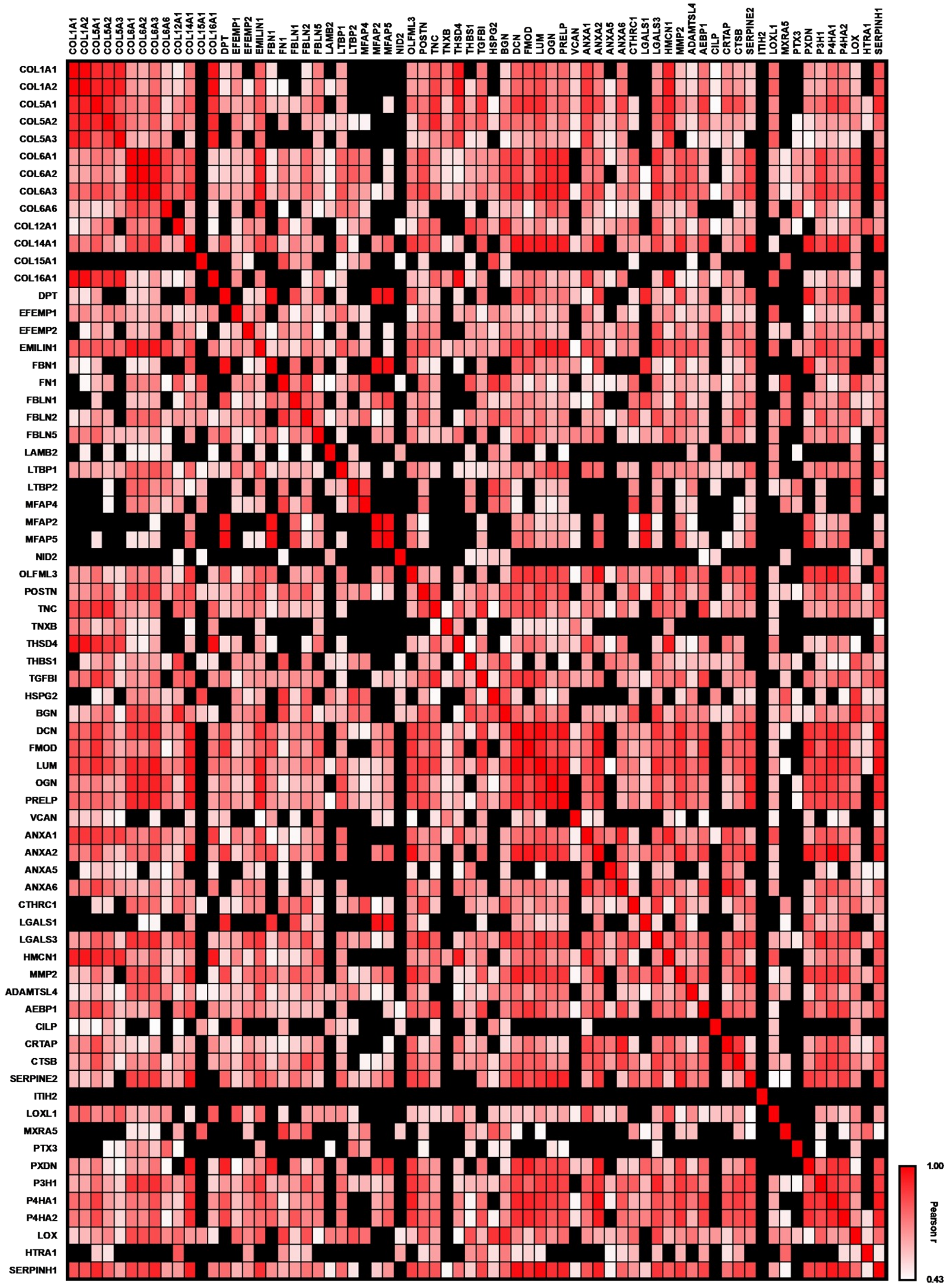

Figure S2. Correlations between all ECM proteins detected within the CAM.

Table S1. List of proteins that is significantly correlate with the donor's age.

Protein Name	Gene Symbol	Pearson r	P-Value
**Donor's age *vs*. Collagens**			
Collagen alpha-2(VI) chain	*COL6A2*	0.55	0.010; **
Collagen alpha-6(VI) chain	*COL6A6*	0.54	0.012; *
Collagen alpha-1(VI) chain	*COL6A1*	0.54	0.012; *
Collagen alpha-3(VI) chain	*COL6A3*	0.50	0.020; *
Collagen alpha-1(XIV) chain	*COL14A1*	0.47	0.031; *
**Donor's age *vs*. Glycoproteins**			
Emilin-1	*EMILIN1*	0.61	0.003; **
Olfactomedin-like protein 3	*OLMFL3*	0.56	0.008; **
Tenascin-X	*TNXB*	0.51	0.019; *
Latent-transforming growth factor beta-binding protein 1	*LTBP2*	0.50	0.021; *
Laminin subunit beta-2	*LAMB2*	0.46	0.035; *
Fibulin-5	*FBLN5*	0.45	0.039; *
**Donor's age *vs*. Proteoglycans**			
Prolargin	*PRELP*	0.61	0.003; **
Mimecan	*OGN*	0.57	0.007; **
Decorin	*DCN*	0.52	0.015; *
Lumican	*LUM*	0.51	0,017; *
Fibromodulin	*FMOD*	0.47	0.034; *
Biglycan	*BGN*	0.46	0.037; *
**Donor's age *vs*. ECM-Affiliated Proteins**			
Annexin A2	*ANXA2*	0.47	0.032; *
**Donor's age *vs*. ECM Regulators**			
ADAMTS-like protein 4	*ADAMTSL4*	0.66	<0.001; ***
Lysyl oxidase homolog 1	*LOXL1*	0.57	0.007; **
Glia-derived nexin	*SERPINE2*	0.53	0.015; *
72 kDa type IV collagenase	*MMP2*	0.49	0.024; *
Prolyl 3-hydroxylase 1	*P3H1*	0.45	0.042; *
Serpin H1	*SERPINH1*	0.43	0.049; *
